# The role of cancer-associated fibroblasts and exosomal miRNAs-mediated intercellular communication in the tumor microenvironment and the biology of carcinogenesis: a systematic review

**DOI:** 10.1038/s41420-024-02146-5

**Published:** 2024-08-26

**Authors:** Reza Nedaeinia, Simin Najafgholian, Rasoul Salehi, Mohammad Goli, Maryam Ranjbar, Hamid Nickho, Shaghayegh Haghjooy Javanmard, Gordon A. Ferns, Mostafa Manian

**Affiliations:** 1 Pediatric Inherited Diseases Research Center, Research Institute for Primordial Prevention of Non-Communicable Disease, Isfahan University of Medical Sciences, Isfahan, Iran; 2 Department of Emergency Medicine, School of Medicine, Valiasr Hospital, Arak University of Medical Sciences, Arak, Iran; 3 Department of Genetics and Molecular Biology, School of Medicine, Isfahan University of Medical Sciences, Isfahan, Iran; 4 Department of Food Science and Technology, Laser and Biophotonics in Biotechnologies Research Center, Isfahan (Khorasgan) Branch, Islamic Azad University, Isfahan, Iran; 5 Advanced Materials Research Center, Department of Materials Engineering, Najafabad Branch, Islamic Azad University, Najafabad, Iran; 6 Department of Immunology, Faculty of Medicine, Iran University of Medical Sciences, Tehran, Iran; 7 Applied Physiology Research Center, Cardiovascular Research Institute, Isfahan University of Medical Sciences, Isfahan, Iran; 8 Brighton and Sussex Medical School, Division of Medical Education, Falmer, Brighton, Sussex UK; 9 Department of Medical Laboratory Science, Faculty of Medical Science Kermanshah Branch, Islamic Azad University, Kermanshah, Iran; 10 Isfahan Neurosciences Research Center, Isfahan University of Medical Sciences, Isfahan, Iran

**Keywords:** Cancer microenvironment, Cancer microenvironment

## Abstract

CAFs (cancer-associated fibroblasts) are highly flexible cells of the cancer microenvironment. They produce the extracellular matrix (ECM) constituents that form the structure of the tumor stroma but are also a source of metabolites, growth factors, chemokines, and exosomes that impact every aspect of the tumor, including its response to treatment. It is believed that exosomal miRNAs facilitate intercellular signaling, which is essential for the development of cancer. The role of miRNAs and CAFs in the tumor microenvironment (TME) and carcinogenesis is reviewed in this paper. The preferred reporting items for systematic reviews and meta-analyses (PRISMA) 2020 guidelines were used to perform a systematic review. Several databases, including Web of Science, Medline, Embase, Cochrane Library, and Scopus, were searched using the following keywords: CAFs, CAF, cancer-associated fibroblasts, stromal fibroblasts, miRNA, exosomal miRNAs, exosome and similar terms. We identified studies investigating exosomal miRNAs and CAFs in the TME and their role in carcinogenesis. A total of 12,572 papers were identified. After removing duplicates (*n* = 3803), 8774 articles were screened by title and abstract. Of these, 421 were excluded from further analysis. It has been reported that if exosomal miRNAs in CAFs are not functioning correctly, this may influence the secretory phenotype of tip cells and contribute to increased tumor invasiveness, tumor spread, decreased treatment efficacy, and a poorer prognosis. Under their influence, normal fibroblasts (NFs) are transformed into CAFs. Furthermore, they participate in metabolic reprogramming, which allows for fast proliferation of the cancer cell population, adaptation to growing energy demands, and the capacity to avoid immune system identification.

## Facts

- CAFs interact with immune cells, cancer vasculature, ECM, and tumor cells in the TME to promote the growth of the tumor by secreting a range of cytokines and chemokines
- CAFs are dynamic cells that play a crucial role in maintaining tumor structural integrity by producing ECM components and secreting factors that affect tumor growth, invasion, and response to treatment.
- The exosomal miRNAs can modulate the behavior of NFs, CAFs, and tumor cells, affecting processes such as conversion from NFs to CAFs, tumor invasiveness, and immune evasion.
- It is well recognized that aberrant expression of exosome-derived miRNAs in CAFs contributes significantly to the growth and spread of cancer.

## Open questions

- Why are CAFs so important in the TME?
- What are CAFs in the TME?
- What is the role of CAFs and exosomal miRNAs in cancer progression?

## Introduction

MicroRNAs are a class of RNAs that are highly conserved and are approximately 25 nucleotides in length; they bind to the 3′ untranslated region (UTR) or open reading frames (ORFs) [1]. This causes gene silencing after the transcription of target mRNAs. miRNAs regulate gene expression in tumors by utilizing multiple signaling pathways. The miRNAs are important for controlling the interaction between CAFs and tumor cells, which affects how tumors grow and develop. Additionally, they can serve as indicators of cancer and objectives for tumor treatment [2, 3].

New insights have emerged concerning the relationship between normal fibroblasts (NFs), CAFs, and cancer cells through exosomal miRNA secretion. Extracellular vesicles can be classified into three primary forms: extracellular vesicles (EVs) are exosomes (~30–150 nm), ectosomes/microvesicles (100–1000 nm), and oncosomes (1–10 µM) [4]. These types of EVs vary in size and biogenesis. Vesicular trafficking, especially exosome-mediated trafficking, has received attention since each of these three vesicles has a significant function in the biology of cancer [5]. According to a recent study, the exosome secretion route is regulated by the Rab family proteins, such as 27a and 27b [6]. It is believed that exosomes facilitate intercellular transmissions, which are essential for cancer growth [7]. Various cell types in the TME secrete them. They are found in all physiological fluids and are taken up by surrounding cells. Exosomes generated from cancer may operate as intercellular messengers by turning microenvironmental cells into tumor-supportive cells by delivering physiologically active chemicals to these recipient cells [8].

**Fig. 1:**
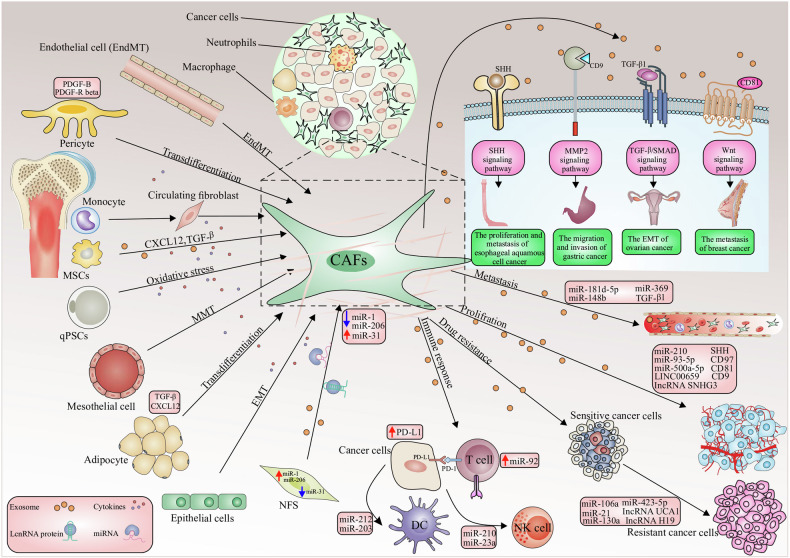
CAFs are mainly derived from local precursor cells that include: mesothelial, bone marrow-derived fibrocytes, epithelial cells, NFs, pericytes, quiescent pancreatic stellate cells (qPSCs), endothelial cells, adipocytes, and mesenchymal stem cells (MSCs). The processes of cell conversion to CAFs consist of the following processes: EMT epithelial-to-mesenchymal transition, EndMT endothelial-to-mesenchymal transition, mesothelial-to-mesenchymal transition MMT. Also, CAF-derived exosomes play a role in the following processes, which include: tumor cell proliferation; conversion of drug-sensitive cancer cells into drug-resistant cancer cells; enhancement of the metastatic capacity of cancer cells; an antitumor immune response by regulating the activity of immune cells. CAF-derived exosomes initiate several molecular processes in tumorigenesis, including TGF-β, to promote EMT, the Wnt signaling cascade contributing to the metastasis of breast cancer, the MMP-2 signaling enhancing the migration and invasiveness of gastric cancer cells. The sonic hedgehog (SHH) signaling pathway thus increases the proliferation and metastasis of ESCC cells.

Exosomal miRNAs can originate from tumor cells or stromal cells such as CAFs [9]. The majority of research has focused on exosomal miRNAs produced from cancer cells [10]. Whilst the exosomal miRNAs produced by CAFs are receiving increasing interest, no studies have looked at their abnormal expression patterns in cancer patients. TME includes both the inherent and external variables that influence the development and dissemination of malignancies. It includes the structural, functional, and metabolic aspects of the tumor tissue, as well as the internal environment within the tumor cells. Tumor cells possess the capacity to impact their own survival and growth circumstances through autocrine and paracrine processes, consequently promoting tumor advancement [11]. The TME and the way that cancer cells interact with their microenvironment depend heavily on CAFs [9]. An essential part of CAF production and activation is played by malignant cells [12]. There are several origins of CAFs including 1- bone marrow cells; 2- mesenchymal stem cells (MSCs); 3- ECs (endothelial cells) and pericytes; 4- resident quiescent normal fibroblasts [13] (Fig. 1). Erdogan et al. claim that the direct interactions between tumor cells and other stromal cells, such as immunological and endothelial cells, cause CAFs to establish a certain biological phenotype [14].

The TME is comprised of endothelial cells, fibroblasts, pericytes, immune cells, stem cells, and bone marrow precursors, as well as the ECM that surrounds them [15]‏. The homeostatic mechanisms that regulate the production and turnover of the ECM, are disturbed in tumors, resulting in the creation of aberrant blood vessels and accumulations of excess fibrillar collagen with a distinct structure [16]. Disrupted ECM homeostasis results in novel forms of paracrine, cell–cell, and cell–ECM interactions, all of which have significant implications for tumor development, angiogenesis, metastasis, immunosuppression, and treatment resistance [15]. The TME is crucial for the growth of CAF cells [17]. Additionally, there is evidence that miRNAs play a significant role in the TME as well as in tumor cells [18]. The complexity and diversity of the TME are gradually being understood through advancements in science[19].

The relationship between cancer cells and the TME is regulated by extracellular miRNAs (ex-miRNAs) and miRNAs. MicroRNAs have been shown to regulate the expression of genes that are expressed post-transcriptionally and to be involved in a wide range of normal and abnormal cellular processes [20]. Recent progress in the assessment of exosomes has made it possible to use miRNAs as biomarkers or therapeutic tools in clinical practice [21]. Mesenchymal stem cells (MSCs) have the potential to differentiate into CAFs when they interact with miRNAs from tumor exosomes within the TME. CAFs can help with several aspects of tumor development, such as growth, EMT, metabolism, invasion, and metastasis. CAFs have the ability to generate pro-angiogenic factors, which can promote angiogenesis and tumor development [22]. CAFs are proliferative, metabolically active cells that resemble fibroblasts and can be implicated in any stage of the development of cancer. According to earlier research, CAFs and tumor cells communicate with one another through the secretion of several chemokines and cytokines that are part of the ECM [23]. Tumor cells can communicate with neighboring cells by secreting soluble molecules into the extracellular space, according to recent research on small extracellular vesicles (sEVs) [24, 25]. Exosomes contain a great variety of bioactive molecules, including signal peptides, microRNAs, lipids, and DNA [26].

Tumor cells release large amounts of exosomes to interact with the environment across extended distances or to deliver paracrine signals [27]. Therefore, by changing the content of vesicles and transporting chemicals to the tumor site that promote the appearance of oncogenic processes such as proliferation, invasion, proliferation of cancer stem cells, and even treatment resistance, exosomal miRNAs can affect carcinogenesis and cancer progression [28].

This review focuses on how exosomal miRNAs influence intercellular interaction and are crucial in the way cancer cells interact with their macro- and microenvironment. Exosomes are a constant carrier of miRNAs. Under different circumstances, miRNAs in the plasma exosome can be maintained. The function of exosomal miRNAs and CAFs in the TME and the development of cancer have been reviewed.

## The study search method

### Methods

Our study followed the guidelines of preferred reporting items for systematic reviews (PRISMA) 2020 guidelines for systematic reviews [29]. To review exosomal miRNAs and CAFs in the TME and carcinogenesis, we searched: Medline, PubMed, Cochrane Library, Embase, Web of Science, and Scopus. We considered studies up until Jan 2023. Additionally, we searched preprint articles on servers like medRxiv and the Social Science Research Network (SSRN). In this search, the following keywords were used: (([Mesh] “MicroRNAs” MicroRNA OR [Handle/Summary] OR miRNAs [Abstract/Title] OR “MicroRNA” [Abstract/Title] miRNA OR [Handle/Summary] OR [Title/Abstract] “Small Temporal RNA” The terms “Tumor-associated fibroblasts” and “Cancer-Associated Fibroblasts” are interchangeable in Mesh. Fibroblasts linked to OR tumors [Title/Abstract] OR “Fibroblasts associated with cancer” [Mesh] OR Fibroblasts associated with cancer [Title/Abstract] OR Fibroblast [Title/Abstract] OR “Fibroblasts” [Mesh] OR “Stromal fibroblasts “[Mesh] Alternatively stated [Title/Abstract])) AND “tumor-associated”[Mesh]“tumor microenvironment “[Mesh] OR Microenvironment *[Introduction/Title] AND (Cancer)[Handle/Summary] OR Carcinogen* [Abstract/Title] Neoplasm OR *[Handle/Summary] Cancer [Handle/Summary] AND (Exosomes*[Title/Abstract] OR (“Exosomes” [Mesh])). To find articles not found by the automated search, a manual search of reference lists and reviews was conducted for potentially eligible articles. We identified duplicate studies and excluded these. We screened the titles and abstracts of the studies and then reviewed the full texts of the articles in two rounds. Two authors, RN and MM, independently studied the two phases. We resolved any disagreements by consulting with RS, the third author.

### Search strategy

#### Study eligibility criteria: inclusion and exclusion

This review focuses on primary research that examined the relationship between exosomal miRNAs and CAFs in the tumor microenvironment and carcinogenesis. This systematic review was conducted using the following criteria for inclusion: original studies published in either English or Persian that have looked into the role of exosomal miRNAs and CAFs across various types of cancer. Based on the PRISMA- 2020 PICO question formulation checklist, which includes P: Population/Disease/Issue I: Intervention, C: Comparison, O: Outcome, and Text, they will assess the article’s suitability. Our focus was on the TME and carcinogenesis as an outcome, but we also looked at the connection between exosomal miRNAs and CAFs in articles where carcinogenesis was not reported. The current review determined the frequency of exosomal miRNAs and CAFs in cancer patients. Case reports, case series, and studies with less than 6 participants, clinical trials, editorials, commentaries, letters to editors, and narrative reviews were excluded. Additionally, we excluded studies that only looked at exosomal miRNAs and CAFs in specific patient populations, such as those with diabetes or dementia, and studies that involved animals or laboratory settings.

### Data extraction

In order to gather articles, eliminate duplicates, and evaluate titles and abstracts, an Endnote library (Version x9) was performed. To begin the process of extracting the data, an expert checklist based on the information gleaned from the articles was created. Only after that was the data extraction carried out. In the studies, information such as the authors’ names, year of publication, country, study design, sample size, age, and gender of patients the study’s specifics, the sample type, the publisher’s nation or area, the detection strategy, and the key findings were collected.

## Results

### PRISMA flow diagram: study selection process

**Fig. 2:**
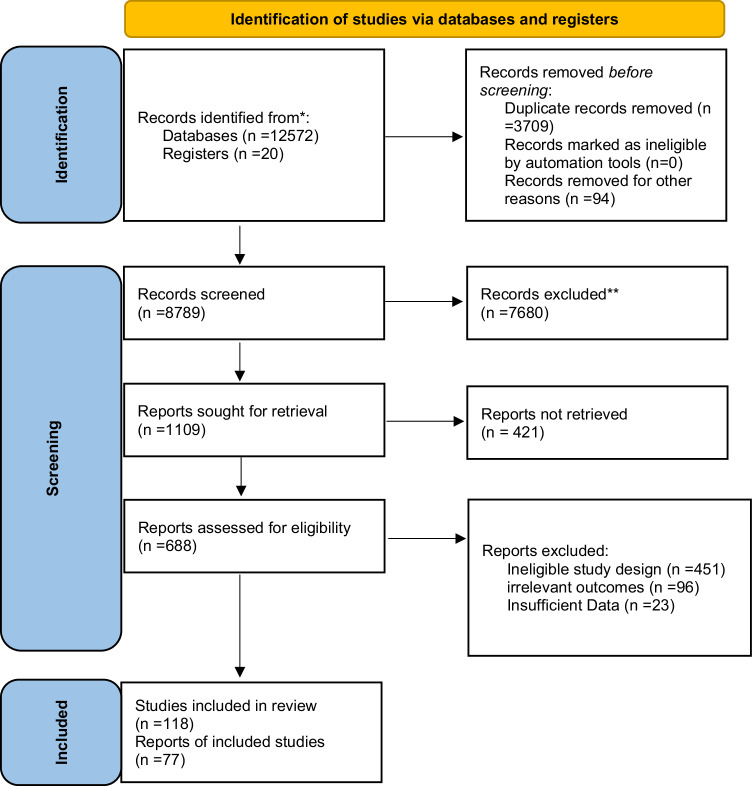
PRISMA flowchart of study selection. *Consider, if feasible to do so, reporting the number of records identified from each database or register searched (rather than the total number across all databases/registers). **If automation tools were used, indicate how many records were excluded by a human and how many were excluded by automation tools.

The study selection process is illustrated in the PRISMA Flow Diagram (Fig. 2), outlining the identification, screening, eligibility, and inclusion of studies in this systematic review. 12,572 studies were identified after searching the different databases. 3709 studies were omitted because they were duplicates. The other 8789 articles were checked for eligibility. After evaluating the full texts, 421 studies out of 1109 with titles and abstracts were not included in the analysis (Fig. 2). Finally, 77 studies listed in Table 1 were included to perform a systematic review.

**Table 1:** Characteristics of included studies that examine exosomal miRNAs and CAFs.

	Authors	Year	Country	Details of study	Sample type	Detection method	Main findings
1	Tao Zhang	2021	China	CAFs-derived exosomes confer cisplatin resistance of NSCLC cells through transferring miRNA-130a and that PUM2 is a critical factor for packaging miRNA-130a into exosomes.	Cell lines	Western Blots Real-Time PCR MiRNA Array Immunofluorescence Assay Cell Transfection Exosome Isolation and Identification	CAFs-derived exosomal miRNA-130a may be a potential therapeutic target for cisplatin resistance in NSCLC.
2	Shan-Shan Yang	2020	China	As a target gene of miR-146a, TXNIP could inhibit the activation of CAFs. miR-146a overexpression or TXNIP silence enhances the activation of the Wnt signal pathway.	Cell lines	PCR and Western blot Cell invasion and migration	Breast cancer (BC)-derived exosomes promote the activation of CAFs through the miR-146a/TXNIP axis to activate the Wnt pathway, which in turn enhances the invasion and metastasis of BC cells.
3	Ashish Kumar	2022	USA	GW4869(chemical compounds) treatment (IP) in mice bearing PC3-R xenografts significantly reduced the tumor weight compared to the vehicle-treated control mice without causing any noticeable toxicity.	Cell lines	Nanoparticle tracking analyses MTT assay Clonogenic assay Tumor Xenograft Western blotting	Inhibiting the release of EVs could sensitize the resistant advanced prostate cancer (PCa) cells to chemotherapy.
4	Yanyin Lu	2021	Japan	Combination method of size exclusion chromatography and concentration filters (SEC-CF) and aimed to characterize different EV types by their size, cargo types, and functions.	Cell lines	EV Fractionation (SEC-CF and PBP (polymer-based precipitation) Western Blotting Fluorescence-Labeling of Cells and EVs EV Molecular Transfer Assay Cell Viability Flow Cytometry	SEC-CF method is useful for the purification of large and small exosomes with higher molecular transfer activities, enabling efficient molecular delivery to target cells.
5	Nicolas Jaé	2015	Germany	Vesicular export mechanism for miR-143, induced by the shear stress-responsive transcription factor KLF2, demonstrates its dependency on Rab7a/Rab27b in endothelial cells.	Cell line	miR degradation assays RNA interference and preparation of extracellular vesicles by-ultracentrifugation qRT-PCR GFP- Immunofluorescence immunoprecipitation	Rab7a/27b dependent, Rab27a-independent, exosomal pathway mediates the export of miR-143.
6	Chunping Wu	2022	China	Dysregulation of exosomal miRNAs derived from cancer‐associated fibroblasts (CAFs) in supraglottic laryngeal squamous cell carcinoma (SLSCC).	Tissues	Reverse transcription-quantitative PCR.	SLSCC patients showed aberrant expression of CAF‐derived exosomal miRNAs. The top five miRNAs and their target genes may jointly constitute a carcinogenic tumor microenvironment and act as biomarkers for SLSCC intervention.
7	Yuan Fang	2019	China	Cancer-associated fibroblasts (CAFs) play vital roles in regulating drug resistance by transferring exosomal miRNA to cancer cells.	Cells	qPCR Western blot analysis Cell proliferation Luciferase reporter assay	CAFs-derived exosomal miR-106b plays a vital role in causing GEM resistance of pancreatic cancer, thus offering a new target for sensitizing pancreatic cancer cells to GEM.
8	Begum Erdogan	2017	USA	CAFs organize the fibronectin matrix through increased contractility and traction forces, which are mediated by MyoII, α5β1-integrin, and PDGFRα. This matrix organization leads cancer cells to migrate directionally using αv integrins	Cells	Fibroblast isolation Co-culture in microfluidic devices Preparation of an FITC-labeled Fn Generation of 3D CDMs and migration assays Calculation of angles between the fibers and FFT analysis Traction-force microscopy Cell-contraction assay Western blot Adhesion turnover assay	New mechanism by which CAFs organize the fibronectin (Fn) matrix and promote directional cancer cell migration.
9	Yuanyuan Li	2021	China	Establish leukemia-associated fibroblasts (LAFs) cell lines from xenografts of HXEX-ALL1, a B-ALL cell line, for that the cell line was established from a 6-years-patient of B-ALL relapsed two times after contemporary risk-directed chemotherapy	Cell line	Cell cycle analysis Colony formation assay Short tandem repeat analysis Immunophenotypic analysis Western blotting analysis Migration assay	HXWMF-1 is the first fibroblastic tumor cell line derived from LAFs or CAFs. In addition, the cell line provided firm evidence that leukemia cells may induce LAFs/CAFs malignant transformation
10	Hua Yin	2021	China	Impact of CAF-derived exosomal miR-135b-5p on CRC progression by targeting thioredoxin-interacting protein (TXNIP).	Cells	RT-qPCR) Western blot analysis Immunofluorescence Flow cytometry Dual luciferase reporter gene assay Angiogenesis detection	TXNIP was confirmed as a target of miR-135b-5p and overexpression of TXNIP could weaken the pro-CRC effect of exosomal miR-135b-5p, And CAF-exosupregulate miR-135b-5p to promote CRC cell growth and angiogenesis by inhibiting TXNIP.
11	Hsin-Jung Wu	2020	USA	Fibroblast-specific inducible focal adhesion kinase (FAK) knockout (cKO) mice in a breast cancer model to study the potential role and mechanisms of FAK signaling in CAF to promote breast cancer metastasis in vivo.	Cell lines	EDU incorporation assay immunohistochemistry Immunoblotting Quantitative PCR miRNA sequencing analysis	New role for FAK signaling in CAFs that regulate their intercellular communication with tumor cells to promote breast cancer metastasis.
12	Jian-Wei Wang	2019	China	Using a miRNA microarray analysis, we identified 13 miRNAs that are significantly increased in exosomes derived from cancer-associated fibroblasts (CAFs) and corresponding paracancer fibroblasts (PAFs)	Cell lines	Western Blot Analysis Scratch Assay Invasion Assay Transfection RT-PCR miRNA Microarray Assays	CAF exosomal miR-1228 is able to promote osteosarcoma invasion and migration by targeting SCAI, which may represent a critical therapeutic target for osteosarcoma treatment.
13	Xiaolan You	2021	China	Mechanism of galectin-1 (GAL-1, encoded by LGALS1) on GC invasion and metastasis in the GC microenvironment.	Cell lines	Lentiviral transduction qRT-PCR Western blotting Wound-healing assay Cell viability assay Cell invasion and migration assays	High expression of GAL-1 in the GC microenvironment predicts a poor prognosis in patients with GC by promoting the migration and invasion of GC cells via EMT through the TGF-β1/Smad signaling pathway.
14	Yao-Yin Li	2018	China	Clarify the role of microRNA encapsulated in the exosomes derived from CAFs in oral squamous cell carcinoma (OSCC).	Tissues	MicroRNA expression transportation of exosomal PCR	The miR-34a-5p/AXL axis confers aggressiveness in oral cancer cells through the AKT/GSK3β/β-catenin/Snail signaling cascade and might represent a therapeutic target for OSCC
15	Ju Hun Yeon	2018	Korea	Our microfluidic EndMT model should foster the analysis and screening of 22 therapeutic candidates for cancer, related to CAFs.	Cells	Microfluidic qRT-PCR	In vitro model to monitor the differentiation of endothelial cells to CAFs via EndMT using a microfluidic device where cellular microenvironment was controlled by applying exosomes and interstitial fluid flow.
16	Tian Fang	2018	China	High-metastatic HCC cells secrete exosomal miR-1247-3p that directly targets B4GALT3, leading to the activation of β1-integrin–NF-κB signaling in fibroblasts.	Tissues	qRT-PCR Microarray analysis Transfected miRNA	Intercellular crosstalk between tumor cells and fibroblasts is mediated by tumor-derived exosomes that control lung metastasis of HCC, providing potential targets for the prevention and treatment of cancer metastasis.
17	S Baroni	2016	Italy	Higher expression of miR-9 in primary triple-negative breast CAFs versus NFs isolated from patients.	Tissues	Quantitative real-time PCR	Role of miR-9 as an important player in the crosstalk between cancer cells and stroma.
18	Xiaocheng Zhou	2018	China	Treatment with exosomes containing overexpressed miR-155 can promote angiogenesis, and the reduction of miR-155 in melanoma cell-secreted exosomes alleviates angiogenesis in vitro and in vivo.	Cells	RT-PCR and Western blot.	Exosomal miR-155 may be a potential target for controlling melanoma angiogenesis and used to set up novel strategies to treat melanoma.
19	Natasha A. N. Jorge	2020	Brazil	Underscore the impact of tumor-microenvironment interactions on disease outcomes and reveal potential non-invasive biomarkers of prognosis and treatment response.	Database	Microarray analysis	Candidate miRNA/target gene pairs with previously reported roles in tumor progression and immune escape mechanisms were further investigated and demonstrated to impact patient’s overall survival not only in melanoma but across different tumor types.
20	Shin La Shu	2018	USA	Human Melanoma-derived exosomes (HMEX) could reprogram human adult dermal fibroblasts (HADF) and cause extracellular acidification.	Cell lines	Western blotting Zetaview Tracking Analysis ExoELISA-ULTRA kit immuno-biochip technology Extracellular flux assays Transfection of miRNA inhibitors	Melanoma-derived exosomes modulate stromal cell metabolism and may contribute to the creation of a premetastatic niche that promotes the development of metastasis.
21	Qiong Li	2013	China	Depletion of miR-21 or the overexpression of Smad7 blocks TGF-β1-induced CAF formation, whereas the overexpression of miR-21 or the depletion of Smad7 promotes CAF formation, even without TGF-β1 stimulation.	Cells	shRNA knockdown Northern blot Real-time PCR	miR-21 and Smad7 are critical regulators of TGF-β1 signaling during the induction of CAF formation.
22	Yuan Zhou	2018	China	Clinical data indicated that a high level of serum exosomal miRNA-21 was correlated with greater activation of CAFs and higher vessel density in HCC patients.	Cell line	RT-PCR Exosome’s tracing	Intercellular crosstalk between tumor cells and HSCs was mediated by tumor-derived exosomes that controlled the progression of HCC.
23	RegallaKumarswamy	2012	Germany	MicroRNA (miR)-21, a microRNA enriched in fibroblasts and involved in general fibrosis, has a role in cardiac EndMT.	Cells	Real-Time-PCR	GF-β-mediated EndMT is regulated at least in part by miR-21 via the phosphatase and tensin homolog/Akt pathway. In vivo, the antifibrotic effects of miR-21 antagonism are partly mediated by blocking EndMT under stress conditions.
24	Jui-Yu Hsieh	2013	Taiwan	smRNA-Seq to WJ-MSCs and BM-MSCs for identifying miRNAs that might regulate the activation of repair-related phenotypes of MSCs, as well as various specific target genes associated with these phenotypes.	Wharton’s jelly- MSCs BM- MSCs	Microarray analysis (RT-qPCR)	miR-146a-5p is critical to the uncoupling of motility and proliferation of MSCs. Our miRNome data also provide a roadmap for further understanding MSC biology.
25	Qiang Li	2018	China	Exosomal miR-21-5p induces MMT and promotes peritoneal metastasis.	Cells	qRT-PCR	Exosomal miR-21-5p induces MMT of PMCs and promotes cancer peritoneal dissemination by targeting SMAD7. Peritoneal mesothelial cells (PMCs) mesothelial-to-mesenchymal transition (MMT)
26	Chi Lam Au Yeung	2016	USA	Significantly higher levels of microRNA-21 (miR-21) isomiRNAs in exosomes and tissue lysates isolated from cancer-associated adipocytes (CAAs) and fibroblasts (CAFs) than in those from ovarian cancer cells.	Tissue, Cell line	Ion Torrent next-generation sequencing technology qRT–PCR in situ hybridization Microarray analysis	Malignant phenotype of metastatic ovarian cancer cells can be altered by miR-21 delivered by exosomes derived from neighboring stromal cells in the omentaltumour microenvironment, and that inhibiting the transfer of stromal-derived miR-21 is an alternative modality in the treatment of metastatic and recurrent ovarian cancer.
27	ShioriWatabe	2020	Japan	Isolated EVs from pleural lavage fluid and focused on EV‐miR‐21 as a diagnostic factor with a relationship to pleural dissemination	Tissue	Microarray In situ hybridization PCR Wound healing assay	EV‐miR‐21 in pleural lavage fluid is important as a diagnostic and prognostic factor. Moreover, EV‐miR‐21 induces the mesenchymal transition (MMT), which can form premetastatic niches of dissemination in the pleural cavity.
28	Jiwoo Lee	2017	Korea	The 3T3-L1 adipocytes indirectly co-cultured with breast cancer cells showed upregulation of inflammation-related genes including Il6 and Ptx3.	Cell lines	mRNA and miRNA expression microarray ELISA Western blot, RT-PCR Luciferase assay Proliferation assay	miRNA-based regulatory mechanism underlying the process of acquiring inflammatory phenotypes in CAA.
29	Shunsuke Yoshii	2019	Japan	Oncogenic role of exosomes derived from TP53‐deficient colon cancer cells in fibroblast proliferation and tumor growth.	Tissue Cell line	Microarray analysis qRT‐PCR RNA interference Cell proliferation and viability Western blot analysis	Cancer cell‐derived exosomes (CDEs) play a pivotal role in tumor progression by fibroblast modification. Cancer cell‐derived exosomes might, therefore, represent a novel therapeutic target in colon cancer.
30	Hongdan Li	2018	China	BM-MSCs play important roles in tumor processes through the release of cytokines or exosomes; however, how BM-MSCs influence the stemness of CSCs in colon cancer cells remains poorly understood.	Cells	MiRNA array analyses Flow cytometric (FCM) analysis Transfections Cell invasion assay Quantitative real-time PCR Western blot analyses	BM-MSC-derived exosomes promote colon cancer stem cell-like traits via miR-142-3p.
31	Guangyao Dai	2018	China	Exosomes containing miR-10b reduced fibroblast proliferation but promoted expression of TGF-β and SM α-actin, suggesting that exosomal miR-10b may activate fibroblasts to become CAFs that express myofibroblast markers.	Cells	miRNA expression profiling array RealtimeqPCR, western blot cell cycle analyses	Olorectal cancer CRC-derived exosomes actively promote disease progression by modulating surrounding stromal cells, which subsequently acquire features of CAFs.
32	J L Hu	2019	China	CAFs-derived molecular determinants that regulate colorectal cancer (CRC) metastasis and chemoresistance have not been fully characterized.	Tissue, Cells	MicroRNA microarray Boyden chamber migration and invasion, cell-counting kit-8, flow cytometry, plate colony formation, sphere formation assay Luciferase report assay, real-time qPCR, western blot, immunofluorescence, and immunohistochemistry	Inhibiting exosomal miR-92a-3p provides an alternative modality for the prediction and treatment of metastasis and chemotherapy resistance in CRC.
33	Damian Medici	2008	USA	Snail and Slug promote the formation of β-catenin–T-cell factor (TCF)-4 transcription complexes that bind to the promoter of the TGF-β3 gene to increase its transcription. Subsequent transforming growth factor (TGF)-β3 signaling increases LEF-1 gene expression causing the formation of β-catenin–lymphoid enhancer factor (LEF)-1 complexes that initiate EMT.	Tissue	RNA Interference Immunocytochemistry, Immunoprecipitation, Immunoblotting PCR Chromatin Immunoprecipitation Invasion/Migration Assays	Β-catenin–LEF-1 complexes can promote EMT without upstream signaling pathways and a unified signaling mechanism driven by the convergence of multiple TGF-β and TCF signaling molecules that confers loss of cell–cell adhesion and acquisition of the mesenchymal phenotype.
34	MojganAhmadzadeh	2008	Bethesda	Addition of TGF-beta1 to the initial Ag activation cultures attenuated the gain of effector function by Ag-specific memory CD8 T cells while the phenotypic changes associated with activation and differentiation into effector memory were comparable to control cultures.	PBMC	Cytokine induction and analysis Flow cytometry analysis RT-PCR	TGF-beta1 suppresses not only the acquisition but also the expression of effector function on human memory CD8 T cells and tumor-infiltrating lymphocytes reactive against melanoma, suggesting that TGF-beta1-mediated suppression can hinder the therapeutic benefits of vaccination, as well as immunotherapy in cancer patients.
35	Valentina R Minciacchi	2017	California	New mechanism of intercellular communication originating from large oncosomes (LO), which are cancer cell-derived, atypically large (1–10 μm) extracellular vesicles (EV)	Cell lines	Immunoblot PKH26 staining Flow cytometry qRT-PCR Tube-branching assay Luciferase reporter assay RNA sequencing Mouse studies Immunoprecipitation	Prostate cancer-derived LO powerfully promotes the establishment of a tumor-supportive environment by inducing a novel reprogramming of the stroma
36	Gyoung Tae Noh	2020	South Korea	Role of exosomal miRNAs from colorectal cancer cell lines in tumorigenesis, by affecting cancer-associated fibroblasts (CAFs), which are vital constituents of the tumor microenvironment.	Cell lines	Immunoblotting culture Flow cytometry migration assay sequencing analysis qRT-PCR	Colon cancer cell lines contained miRNA let-7d in secreted exosomes targeting the chemokine CCL7. Exosomes from colorectal cancer cell lines affected CCL7 secretion from CAFs, possibly via the miRNA let-7d, and interfered with the migration of CCR2+ monocytic THP-1 cells in vitro.
37	Alexandra Fullár	2012	Austria	Co-culture of periodontal ligament (PDL) fibroblasts and SCC-25 oral squamous carcinoma cells (OSCC), results in the conversion of PDLs into carcinoma-associated fibroblasts (CAFs).	Cell lines	RT-PCR Immunocytochemistry ELISA	Fibroblast-produced inactive MMP-2 has been activated by the tumor-cell-produced membrane-type 1 matrix metalloproteinase (MT1-MMP). The crosstalk between cancer- and the surrounding fibroblast stromal cells is essential for the fine-tuning of cancer cells invasivity.
38	Akira Orimo	2005	USA	Carcinoma-associated fibroblasts (CAFs) extracted from human breast carcinomas promote the growth of admixed breast carcinoma cells significantly more than normal mammary fibroblasts derived from the same patients.	Tissue	Real-time PCR Flow Cytometry Cell Proliferation Assay	Fibroblasts within invasive breast carcinomas contribute to tumor promotion in large part through the secretion of SDF-1.
39	NetaErez	2010	Switzerland	That performed expression profiling of fibroblasts from dysplastic skin of K14-HPV16 transgenic mice and investigated the functional implications of tumor-promoting inflammation mediated by CAFs.	Cell line	Transgenic Mice and Human Samples Orthotopic Tumors	Normal dermal fibroblasts can be “educated” by carcinoma cells to express proinflammatory genes.
40	Elisa Giannoni	2010	Italy	Using fibroblasts from human patients with benign prostatic hyperplasia or aggressive carcinoma, and identify a circuitry in which cancer cell–produced interleukin-6 (IL-6) affects fibroblast activation.	Cells	Immunoprecipitation and Western blot analysis In vitro Boyden invasion assay Flow cytometry Xenograft experiments Immunohistochemical analysis	CAF-induced EMT leads prostate carcinoma cells to enhance the expression of stem cell markers, as well as the ability to form prostaspheres and to self-renew. Hence, the paracrine interplay between CAFs and cancer cells leads to an EMT-driven gain of cancer stem cell properties associated with aggressiveness and metastatic spread.
41	Maria-GiuseppinaProcopio	2016	USA / Switzerland	CSL silencing induces senescence of primary fibroblasts from the dermis, oral mucosa, breast, and lung. CSL functions in these cells as a direct repressor of multiple senescence- and CAF-effector genes.	Tissue and cells	Immunodetection techniques MicroscaleThermophoresis DNA-oligo pull-down assay Transcriptome analysis ChIP and ChIP-Seq	Concomitant loss of CSL and p53 overcomes fibroblast senescence, enhances expression of CAF effectors and promotes stromal and cancer cell expansion. The findings support a CAF activation-stromal co-evolution model under convergent CSL-p53 control.
42	Hikaru Sugimoto	2006	USA	FSP1 is a unique marker of fibroblasts with marginal overlap with other markers such as αSMA, suggesting for the first time that CAFs represent a heterogeneous population.	Cell line	Mice model Immunohistochemistry Morphometric analysis	Tumor microenvironment associated fibroblasts are a heterogeneous population and thus, the use of αSMA or vimentin as the only markers will not identify all the CAFs.
43	Jean Albrengues	2015	France	In human carcinomas from different origins, LIF induces a sustained proinvasive activation of CAF through an epigenetic-dependent loss of SHP-1 phosphatase.	Cell	RNAi transfections invasion assays Western blots and Co-immunoprecipitation analysis RT–qPCR analysis DNMT activity assay Bisulfite sequencing	Combined inhibition of DNMT activities and JAK Signaling, in vitro and in vivo, results in long-term reversion of CAF-associated proinvasive activity and restoration of the wild-type fibroblast phenotype.
44	Fernando Calvo	2015	UK	Cdc42EP3/BORG2 is required for the matrix remodeling, invasion, angiogenesis, and tumor-growth-promoting abilities of CAFs. Cdc42EP3 functions by coordinating the actin and septin networks.	Cell lines	Immunofluorescence Western Blotting qRT-PCR	Cdc42EP3 sensitizes fibroblasts to further cues-in particular, those activating actomyosin contractility and thereby enables the generation of the pathological activated fibroblast state. Cdc42EP3 as a key regulator of the conversion of normal fibroblasts into CAFs. CAFs have enhanced stress fibers, αSMA-positive fibers, and focal adhesions
45	Bi‐Lan Li	2018	China	Significant decrease of miR‐148b in CAFs and CAFs‐derived exosomes and miR‐148b could be transferred from CAFs to endometrial cancer cell through exosomes.	Tissue, Cells	qRT‐PCR Migration and invasion assays Luciferase reporter assay Western blot analysis In vivo experiments	CAFs‐mediated endometrial cancer progression is partially related to the loss of miR‐148b in the exosomes of CAFs and promoting the transfer of stromal cell‐derived miR‐148b might be a potential treatment to prevent endometrial cancer progression.
46	Na Zhang	2020	China	Clinical significance and biological function of EV encapsulated miR-320a released from CAFs in EC.	Cells	Microarray analysis Western blot analysis RT-qPCR Flow cytometry	Due to the downregulation of HIF1α by miR-320a, which led to lowered VEGFA expression in vitro. Accordingly, we overexpressed HIF1α also showed that the inhibitory effect of miR-320a overexpression in EC cells could be reversed. These results point to CAF-derived EVs carrying overexpressed miR-320a as a novel direction for therapeutic strategies for EC.
47	Aki Miyasaka	2015	Japan	Investigate the roles of TP53 and MAPK/PI3K pathways in endometrial carcinomas and to identify appropriate radiosensitizing therapeutics.	Cell lines	Clonogenic assays qRT‐PCR Luciferase reporter assay	TP53 mutation and PI3K pathway activation enhances radioresistance in endometrial carcinomas and that targeting the PI3K/mTOR or HIF1α pathways could improve radiosensitivity.
48	Guang Shan	2021	China	Role of CAF-derived exosome miR-148b-3p in bladder cancer progression and chemosensitivity.	Tissue	Flow cytometry Western blot analysis transwell invasion assay qRT-PCR Cell transfection Dual luciferase reporter assay	miR-148b-3pdownregulation in CAF-derived exosomes, thereby inhibiting the Wnt/β-catenin pathway and promoting PTEN expression, may offer potential opportunities for bladder cancer treatment.
49	Yonglei Liu	2020	China	miR-3613-3p was upregulated in exosomes from fibroblasts educated by TGF-β1 and the fibroblasts from breast cancer tissues. Exosomal miR-3613-3p promoted breast cancer cell proliferation and metastasis.	Cell lines	Western blotting miRNA array Real-time RT-PCR Lentivirus carrying miRNA Cell invasion ROS detection Luciferase assay	Activated fibroblasts exosomes with high levels of miR-3613-3p played an oncogenic role in breast cancer cell survival and metastasis, which suggested that miR-3613-3p functions as a therapeutic target.
50	Qinli Zhao	2020	China	Identify and evaluate serum exosomal miRNAs for LSCC diagnosis.	Serum	Exosome Isolation by ExoQuick (EQ) Solution Western blot and Antibody Array Bioinformatics Analysis of RNA-seq Data qRT-PCR Transfection Cell Invasion Assay	Serum exosomal miR-941 may serve as a promising oncogenic biomarker for diagnosing LSCC and has the potential as a therapeutic target.
51	Jingting Wang	2014	China	Prognostic and diagnostic values of exosomal ncRNA by comparing the amounts of Exosomal miR-21 and HOTAIR in serum of laryngeal squamous cell carcinoma (LSCC) patients with those of polyps of vocal cords, and by determining whether combined detection of the two molecules could provide useful information in the diagnosis of LSCC.	Peripheral blood	Western blot analysis qRT-PCR	Serum exosomal miR-21 and HOTAIR were significantly correlated with clinical parameters of LSCC, and the combined evaluation of their serum expressions may be a valuable biomarker to screen LSCC and might be a promising predicting tool for LSCC patients.
52	Yong Qi	2022	China	Effect of LIMA1 and exosome-associated miR-20a-5p in HCC development. LIMA1 and miR-20a-5p expression levels	Tissue, Cells	Real-time quantitative PCR (qRT-PCR), western blotting, or immunohistochemistry (IHC). 5-ethynyl-2′-deoxyuridine (EdU) assays, colony formation assays, wound-healing assays, and Transwell invasion assays	IMA1 is a tumor suppressor that inhibits the Wnt/β-catenin signaling pathway and is downregulated by CAF-derived exosomes carrying oncogenic miR-20a-5p in HCC.
53	Daniela Likonen	2022	Israel	Exosomal telomerase might play a role in modifying NFs into CAFs by upregulating αSMA and Vimentin, two CAF markers.	Cell lines	Cell’s transfection Q-real-time PCR reaction Western blot ELISA assay microRNA analysis Cell cycle analysis enrichment analysis	By ectopically expressing microRNA 342, one of the top identified microRNAs shows that it may mediate the proliferative phenotype that these cells acquire upon taking-up exosomalhTERT, providing them with a survival advantage.
54	Cassandra RinguetteGoulet	2018	Canada	TGFβ inhibitors attenuated CAF marker expression in healthy fibroblasts. Therefore, these data demonstrate that bladder cancer cells trigger the differentiation of fibroblasts to CAFs by exosome-mediated TGFβ transfer and SMAD pathway activation.	Cell	CAF induction Cell proliferation assay Immunoblot	New function for bladder cancer exosomes as novel modulators of stromal cell differentiation.
55	Ssu-Chuan Lai	2019	Taiwan	Underlying mechanism regarding the interplay between DNMTs and IL-6-induced OCT4 expression and the sorafenib resistance of HCC remains largely unclear.	Cell lines	ELISA assay qRT-PCR Immunoblotting Aldehyde dehydrogenase (ALDH) activity assay Tumor xenograft mouse model Immunohistochemical Cell viability assay	Highlight the significance of IL-6-DNMT3b–mediated OCT4 expressions in future therapeutic target for patients expressing cancer stemness-related properties or sorafenib resistance in HCC.
56	Claire Vennin	2019	Australia, UK	Heterogeneous subtypes of CAFs coexist within pancreatic cancer tissues and can both promote and restrain disease progression.	Cell lines	Immunoblotting Organotypic assays Second Harmonic Generation (SHG) imaging and analysis Grey-level co-occurrence matrix Real-time quantitative polymerase chain reaction cell-derived matrix-establishment and monitoring of cell streaming Microarray transcript expression profiling GSEA analysis QuPath-based quantification of DAB staining in GEMMs Proteomics analysis Generation of KO and KRAB lines with CRISPR-Cas9 Magpix analysis of canonical NF-kB signaling Implantation of titanium window for longitudinal imaging FLIM-FRET imaging and analysis	Depleting perlecan in the stroma combined with chemotherapy prolongs mouse survival, supporting it as a potential target for anti-stromal therapies in pancreatic cancer.
57	Lin Shi	2020	China	Ability of microRNA‐20a (miR‐20a) within these CAF‐derived exosomes to influence non‐small‐cell lung cancer (NSCLC) progression.	Tissues and Cells	Western blotting, nanoparticle tracking analyses, and transmission electron microscopy. qPCR and fluorescence in situ hybridization (FISH). CCK‐8, EdU uptake, and colony formation assessments	CAFs can release miR‐20a‐containing exosomes capable of promoting NSCLC progression and chemoresistance, highlighting this pathway as a possible therapeutic target in NSCLC.
58	Naho Fujii	2023	Japan	Effects of exosomes derived from cancer-associated fibroblasts (CAFs) on the proliferation of malignant melanoma (MM) cells and evaluated their clinicopathological significance.	Cell lines	Western blotting MTT assays immunohistochemically	CD9 expression in CAFs is a promising prognostic marker for patients with malignant melanoma.
59	Cindy Neuzillet	2022	France	Combination of both (periostin) POSTN‐positive CAFs and (podoplanin)PDPN‐positive CAFs was associated with specific features of the microenvironment (in terms of stromal abundance and immune cell infiltrates) and prognosis, which suggests cooperation between CAF subpopulations in PDAC (Pancreatic ductal adenocarcinoma).	Tissue and Cells	Immunohistochemistry Multiplex immunofluorescence RNAseq analysis	Periostin-positive CAF is an early, activated CAF, associated with aggressive tumors, whereas a podoplanin‐positive CAF is associated with an immune‐related phenotype. These two subpopulations cooperate to define specific tumor microenvironment
60	Guangyue Luo	2021	China	Mechanisms of CAF-derived exosomal LINC00355 in bladder cancer (BC) cell resistance to cisplatin.	Cell lines	MTT proliferation assay and flow cytometric analysis Transfection and Luciferase activity assay qRT-PCR and western blot	Exosomal LINC00355 derived from CAFs promotes BC cell resistance to cisplatin by regulating the miR-34b-5p/ABCB1 axis.
61	Hua Guo	2019	China	Explore the function of cancer-associated fibroblast (CAF)-derived exosomal microRNA-98-5p (miR-98-5p) in cisplatin resistance in OC and the participation of CDKN1A.	Cells	RT-qPCR and western blot analysis Dual luciferase reporter assay CCK-8, colony formation, and flow cytometry assays	CAF-derived exosomes carrying overexpressed miR-98-5p promote cisplatin resistance in OC by downregulating CDKN1A.
62	Xing Qin	2019	China	Molecular mechanisms by which exosomal miR-196a modulate cisplatin resistance in HNC (Head and neck cancer) cells.	Cells	Western blot analysisMTT assay Fluorescent labeling and transfer of exosomesCell transfection Biotin miRNA pull-down assay real-time PCR analysis (RNA immunoprecipitation) RIP assaytumorigenicity assay in vivo	CAF-derived exosomal miR-196a confers cisplatin resistance in HNC by targeting CDKN1B and ING5, indicating miR-196a may serve as a promising predictor of and potential therapeutic target for cisplatin resistance in HNC.
63	Haiyang Zhang	2020	China	Role of CAFs in regulating lipid metabolism as well as ferroptosis of cancer cells is still unexplored and remains enigmatic.	Cells	Mass spectrum RT-qPCR Mitochondrial membrane potential western blotting biotin miRNA pull-down assay Immunohistochemistry	CAFs secrete exosomal miR-522 to inhibit ferroptosis in cancer cells by targeting ALOX15 and blocking lipid-ROS accumulation.
64	Katherine E. Richards	2017	Indianapolis, IN	Treatment of gemcitabine-exposed CAFs with an inhibitor of exosome release, GW4869, significantly reduces survival in co-cultured epithelial cells, signifying an important role of CAF exosomes in chemotherapeutic drug resistance.	Cell lines	Lentivirus Transduction RT-PCR Western Blot	Potential for exosome inhibitors as treatment options alongside chemotherapy for overcoming PDAC chemoresistance.
65	Xiaoping Chen	2023	China	Exosomes of CAFs originating from human ovarian cancer hindered tumor cell proliferation, metastasis, and EMT in vitro.	Tumor tissue	Validation of circular structure and head-to-tail splicing of circRNA RNA fluorescence in situ hybridization (FISH). Cell transfection. Cell proliferation capacity detection. Transwell assay. Dual luciferase reporter assay. Biotin miRNA pull-down assay. Western blot assay. qRT-PCR.	Previously unknown regulatory pathway whereby CAFs-derived exosomes delivered circIFNGR2 and inhibited the malignant progression of OVCA by circIFNGR2/miR-378/ST5 axis.
66	Stephanie Hunter	2019	Canada	Roles of cyclo-oxygenase (COX)-2 induced miR-526b and miR-655 in tumor-associated angiogenesis and lymphangiogenesis.	Cell line	Tube Formation Assay HUVEC Migration Assay qRT-PCR Western Blot	Strengthen the role of miRNAs as breast cancer biomarkers and *EP4* as a potential therapeutic target to abrogate miRNA-induced angiogenesis and lymphangiogenesis in breast cancer.
67	Er-Bao Chen	2019	China	Roles of dysregulation of micro(mi)RNAs and NK cells in the progression of hepatocellular carcinoma (HCC).	Cell lines	qRT-PCR Animal Subjects smRNA-seq Tumor Growth and Pulmonary Metastasis In Vivo Assays	MiR-561-5p/CX3CL1/NK cell axis that drives HCC metastasis and demonstrated that CX3CR1+NK cells serve as potent antitumor therapeutic effectors.
68	Yunyun Liu	2019	China	Downregulation of miR-340-5p in GBM was correlated with tumor size, recurrence, and poor survival. miR-340-5p levels correlated with the density of TAMs and M2-polarized TAMs in GBM	Cell lines	In situ hybridization (ISH) Immunohistochemistry (IHC), Immunofluorescence (IF), Migration assay, Flow cytometry, Xenograft tumor assay, Bioluminescent imaging and analysis, Lentivirus packaging and transduction Luciferase reporter assay, Chromatin immunoprecipitation (ChIP) assay, Western blotting and RT-PCR	MiR-340-5p-macrophage feedback loop modulates the progression and tumor microenvironment of GBM and may represent a prognostic biomarker and therapeutic strategy for GBM.
69	Shuo Wang	2022	China	Exosomes (NFs-Exo and CAFs-Exo) were then isolated from the supernatant of NFs and CAFs. Next, the differentially expressed miRNAs (DEMs) between NFs-Exo and CAFs-Exo were identified using RNA-sequencing.	Cells	Western blot assay (RNA-seq) Cell-counting Kit-8 (CCK-8) -assay Transwell assays RT-qPCR Luciferase reporter and TOPflash reporter assays RNA pull-down assay (IHC)	Exosomal miR-1290 from CAFs could promote PC cell growth and metastasis via inhibiting GSK3β/β-catenin signaling, suggesting that miR-1290 may serve as a potential therapeutic target for the treatment of prostate cancer.
70	Matthew Kraman	2010	UK	Depletion of FAP-expressing cells, which made up only 2% of all tumor cells in established Lewis lung carcinomas, caused rapid hypoxic necrosis of both cancer and stromal cells in immunogenic tumors by a process involving interferon-γ and tumor necrosis factor-α.	Cells	Transgenic mouse IHC immunofluorescence	FAP-expressing cells are a nonredundant, immune-suppressive component of the tumor microenvironment.
71	Ana Costa	2018	France	Four CAF subsets in breast cancer with distinct properties and levels of activation. Two myofibroblastic subsets (CAF-S1, CAF-S4) accumulate differentially in triple-negative breast cancers (TNBC)	Cells	Flow Cytometry Immunohistochemistry (IHC) Triple-Immunofluorescence RNA Sequencing qRT-PCR Silencing Experiments Using Small-Interfering RNA Transwell migration assay	In contrast to CAF-S4, CAF-S1 enhances the regulatory T-cell capacity to inhibit T effector proliferation. These data are consistent with FOXP3+T lymphocyte accumulation in CAF-S1-enriched TNBC and show how a CAF subset contributes to immunosuppression.
72	Christine Feig	2013	United Kingdom	The depletion of the FAP+ stromal cell also uncovered the antitumor effects of α-CTLA-4 and α-PD-L1, indicating that its immune-suppressive activity accounts for the failure of these T-cell checkpoint antagonists.	Cell lines	ELISpot Assays. Immunofluorescence. Immunohistochemistry Flow Cytometry RNA-Sequencing	The residual tumor was composed only of premalignant epithelial cells and inflammatory cells. Thus, a single protein, CXCL12, from a single stromal cell type, the FAP+CAF, may direct tumor immune evasion in a model of human PDA.
73	Ivy X Chen	2019	Cambridge, MA	Immunosuppressive effects are dependent on CXCR4 signaling in αSMA+ cells, which include cancer-associated fibroblasts as well as other cells such as pericytes.	Cells	Bioinformatics Analysis of RNA-seq Data And Microarray knockout studies	CXCL12/CXCR4-mediated desmoplasiain metastatic breast cancers promotes immunosuppression and is a potential target for overcoming therapeutic resistance to immune checkpoint blockade in Metastatic breast cancer patients.
74	Tang X	2016	China	miR-200s and their targets influenced collagen contraction by CAFs. The upregulation of fibronectin and lysyl oxidase directly by miR-200 or indirectly through Fli-1 or TCF12 contributed to ECM remodeling, triggering the invasion and metastasis of breast cancer cells both in vitro and in vivo.	Tumor tissues	qRT-PCR Western blot Enzyme-linked immunosorbent assay Luciferase reporter assay Wound-healing and invasion assays Collagen-remodeling assay IHC, immunofluorescence Orthotopicxenografts and lung metastasis analysis	Role of CAF activation and function in the promotion of cancer cell invasion through ECM remodeling and provide a considerable amount of information that will be useful for the development of stromal therapeutic targets.
75	Baroni S	2016	Italy	Tumor-secreted miR-9 can be transferred via exosomes to recipient NFs and this uptake results in enhanced cell motility. Moreover, this miRNA is also secreted by fibroblasts and in turn able to alter tumor cell behavior, by modulating its direct target E-cadherin, and NFs themselves.	Tumor tissues and cells	Real-time PCR Migration, wound-healing and invasion assays western blot analysis Gene expression profiling Tumor growth analysis in orthotopicxenografts	Capability of NFs transiently transfected with miR-9 to promote in vivo tumor growth. Taken together, these data provide new insights into the role of miR-9 as an important player in the crosstalk between cancer cells and stroma.
76	S. Khazaei	2016	Iran	miR-451 expression in serum and tissue samples of esophageal squamous cell carcinoma (ESCC) patients.	Tissues	RT-PCR Migration assay Analyzing MIF expression in the co-cultured cell lines	Cancer-associated fibroblasts use exosomal miR-451 as a signaling molecule to provide a favorable niche for tumor cell migration and cancer progression.
77	Jingya Wang	2018	China	High level of miR-27a in exosomes derived from GC cells. miR-27a was found to function as an oncogene that not only induced the reprogramming of fibroblasts into CAFs but also promoted the proliferation, motility, and metastasis of cancer cells in vitro and in vivo.	Cell line	Real-time PCR, western blotting, cell proliferation, migration, invasion and wound-healing assays, Immunofluorescence miRNA microarray analysis, Cytokine Array, Luciferase assay	miR-27a in exosomes derived from GC cells has a crucial impact on the microenvironment and may be used as a potential therapeutic target in the treatment of GC.

### Potential role of exosomal miRNAs in the colonization of metastasized CAFs cells

Exosomes and CAFs have important functions in the TME. Exosomes, which are vesicles generated by tumor cells outside of the cells, contain proteins that can interact with immune cells and hinder their capacity to kill tumors, thereby facilitating the evasion of cancer by the immune system. Conversely, CAFs play a crucial role in inhibiting the immune response within the TME, hence promoting tumor invasion and dissemination. Both exosomes and CAFs play a role in creating an immunosuppressive environment in the TME [30].

Because of the distribution of exosomes to neighboring cells, CAFs play a critical role in regulating the formation of malignancies [31]. Drug resistance, angiogenesis, invasion, metastasis, and tumor development are all significantly influenced by exosomal miRNAs. They control mechanisms that affect the microenvironment and cancer immunity. Two examples of the endosomal sorting complex required for transport (ESCRT)-containing proteins that are in charge of exosome formation are Alix (Apoptosis-linked gene 2-interacting protein X) and Tsg101 (Tumor susceptibility gene 101 protein). Proteomic investigations of isolated exosomes from various cell types have found Tsg101, Alix, and ubiquitinated proteins [32].

Recent studies have shown that cancer cells generate more exosomes than normal cells, even in the very early phases of carcinogenesis. Analysis of proteomic profiling of blood-circulating extracellular vesicles in breast cancer holds great promise for early detection and diagnosis [33]. Tumor-derived exosomes (TEX) are exosomes generated by tumors that are crucial for managing tumor cells inside the TME. It was discovered that TEXs were significant even in the pre-cancer stage. Furthermore, TEXs with physiologically active miRNAs can mirror the unique features of different types of cancer, impacting tumor growth. Induction of the TEM from melanoma cells [34], transfer of miRNAs supporting 1-integrin-NF-kB signaling from metastatic liver cancer cells [35], improvement of breast cancer cell motility and ECM remodeling pathways [36], and activation of SOCS1/JAK2/STAT3 signaling from melanoma cells [37] (Fig. 1). According to a recent study, the presence of miRNAs in TEXs may help tumors survive by changing NFs into CAFs. In human melanoma cells,miR-21, miRNA-211, miR-222, miR-214, miR-155, and miR-210 may enhance the activation of NFs to undergo CAF transformation [38]. Consequently, there may be a considerable shift in the CAF gene’s expression [39]. It is thought that miR-21 is the primary regulator of oncogenic processes. It enhances cell survival, CAF formation, and activation by regulating TGF-β1 signaling [40]. Healthy hepatic stellate cells were converted into CAFs by isolated exosomes from patients with hepatocellular carcinoma (HCC) via miR-21, which downregulated PTEN (Tumor suppressor gene) and activated the PI3K/AKT signaling pathway [41].

miR-21 has been reported in several types of tumors. The exosomal miR-21-5p released by cancer cells promotes angiogenesis and it is involved in neoplastic processes [42]. An interesting point is related to the promotion of secretion of angiogenic factors through exosomal miR-21 released from cancer cells and its role in neoplastic processes [43]. MiR-21 has been shown to activate NFs and produce the CAF phenotypic markers SMA and S100A4, respectively in studies from colorectal and malignant esophageal cancer cells [39]. The two most common miRNAs, miR-21 and miR-146a, which are recognized as significant regulators of CAF formation, were involved in this process [44, 45]. Furthermore, by stimulating the expression of transforming growth factor-β (TGF-β)/ Smad pathway, which can cause endothelial cells (ECs) to convert into CAFs (EMT), exosomal miR-21-5p induces EMT in gastric cancer (GC) [46] (Fig. 1). Next-generation sequencing technology has shown that the exosomal transfer of miR-21 from CAFs to OVCA (Ovarian cancer) cells inhibited apoptosis and increased resistance to treatment with paclitaxel by reducing the expression of apoptotic peptidase activating factor (APAF1) [47].

When exosomal miR-21-5p causes the peritoneal cavity to undergo mesothelial–mesenchymal transition (MMT), lung cancer is more likely to spread [48]. Mesothelial cells have been identified as a source of CAFs in peritoneal carcinomatosis (PC) [49]. Therefore, the exosomal interaction between tumor cells, CAFs, and tumor-associated macrophages (TAMs) is a major factor in the growth of malignancies [50]. While miR-10b, miR-6819-5p, miR-6737-5p, and miR-1249-5p have been shown to have indirect oncogenic activity via controlling fibroblast function [51–53]. CAFs release exosomal miR-1228, which has the ability to enhance the invasion and migration of osteosarcoma by targeting suppressor of cancer cell invasion (SCAI). Exosomal collagen type VI alpha 1 (COL6A1), transforms healthy fibroblasts into CAFs, facilitating the invasion and migration of osteosarcoma (OS) cells via the TGF-β/COL6A1 signaling pathway [54, 55]. In the context of OS, studies have shown that OS cell-derived exosomal components, such as COL6A1 and leukemia inhibitory factor receptor antisense RNA1 (LIFR-AS1), can induce the transformation of NFs into CAFs. Once activated, these CAFs can enhance the migration and invasion of OS cells through the secretion of proinflammatory cytokines and interaction with microRNAs. Additionally, the interaction between CAF-secreted exosomes and OS cells can promote malignancy grade and contribute to the development of OS. Moreover, CAFs contribute to immune evasion and tumor progression by modulating the immune microenvironment, promoting the differentiation of myofibroblasts, and enhancing angiogenesis [55]. Furthermore, CAF-derived exosomes have been implicated in promoting angiogenesis within the TME, which is a crucial process for tumor growth. These exosomes can deliver pro-angiogenic factors and signaling molecules to endothelial cells, stimulating the formation of new blood vessels to support the growing tumor. Additionally, CAF-derived exosomes have been associated with immunosuppressive effects, influencing the immune response within the TME and facilitating immune escape by cancer cells [54].

### To what extent can the presence of CAFs affect the development of cancer?

Numerous cell processes, including tumor development, EMT, metabolism, metastasis, and invasion can all be accelerated by CAFs [56]. The EMT plays an important role in both carcinogenesis and embryogenesis. EMT is mostly responsible for the invasion of single cells, which modifies the morphology and adhesion characteristics of cancer cells and represses epithelial markers in favor of mesenchymal signatures. The expression of genes associated with EMT may be stimulated by the interaction between β-catenin accumulation and T-cell factor/lymphoid enhancer factor (TCF/LEF), a crucial EMT initiator [57]. A study has demonstrated that the high heterogeneity of CAFs allows them to exhibit two functionalities in disease: while CAFs can promote angiogenesis, inflammation, immunosuppression, and metastasis, which ultimately lead to the growth of tumors, the antitumor characteristics of fibroblasts contribute to processes such as ECM production, the growth and differentiation of epithelial cells, and the response to tissue damage [58]. CAFs can also produce a variety of growth factors and proinflammatory cytokines, such as TGF-β, vascular endothelial growth factor(VEGF), interleukin-6 (IL-6), and CXC chemokine ligand 12 (CXCL12), to support angiogenesis and attract immunosuppressive cells into the TME to aid in immune evasion [59]. Furthermore, CAFs can contribute to cancer development indirectly (via soluble factors and miRNA) as well as directly (through intercellular communication). Exosomes generated by CAFs control the TME and mediate the growth and dissemination of cancer cells [56]. Numerous bioactive substances, including DNA, lipids, signal peptides, and miRNAs, are carried by exosomes. Among these molecules are exosomal miRNAs, which are important regulators of the TME. It is well recognized that aberrant expression of exosome-derived miRNAs in CAFs contributes significantly to the growth and spread of cancer [60] for example, miR-9 and miR-200s induce NFs in TME to transform into CAFs and promote tumor metastasis [36, 61].

CAFs interact with immune cells, cancer vasculature, ECM, and tumor cells in the TME to promote the growth of the tumor by secreting a range of cytokines and chemokines. Proliferative signaling is aided by a number of CAF-derived substances that help cancer cells evade growth suppressors and withstand cell death. Exosomes from the CRC cell lines HT-29 and SW480, perhaps via miRNA let-7d, inhibited the migration of CC-type chemokine receptor 2 (CCR2) monocytes (THP-1 cells) and the release of chemokine (C-C motif) ligand 7 (CCL7) from CAFs in vitro [62]. According to Fullár et al., membrane-type 1 matrix metalloproteinase (MT1-MMP), which is generated by tumor cells, activates inactive matrix metalloproteinase-2 (MMP-2) produced by fibroblasts. The fine-tuning of cancer cell invasion is dependent on the activated MMP-2 [63]. In oral squamous cell carcinoma (OSCC) is further increased by the miR-34a-5p/AXL axis. The AKT/GSK3β/β-catenin signaling pathway facilitates this process. This may trigger EMT, which raises the metastasis of cancer cells [31]. MiR-451 is a tumor suppressor that is regulated in various types of tumors. In contrast, CAFs use small molecules called exosomal miR-451 to signal and help cancer cells move and grow [64]. Furthermore, it has been shown that the miRNA levels in CAFs and cancer cells may vary differ from those in exosomes [9]. Tumor stroma has been demonstrated to have a large distribution of CAFs. Myofibroblasts and fibroblasts combine to become CAFs [65]. It is well known that these CAFs stimulate angiogenesis, which contributes to tumor development and, consequently, to the growth and spread of cancer cells [66] for example miR-526b and miR-655 promote angiogenesis and lymphangiogenesis in TME [67].

Recent studies suggest that unregulated epigenetic control of gene expression and metabolic adaptive response may be the cause of the aberrant activation of CAFs [68]. Regular fibroblasts (NFs) are not like activated CAFs; instead, they change cell surface markers. During carcinogenesis, CAFs’ pro- and antitumorigenic activities are probably dynamic. For example, in order to enhance the CAF phenotype in breast cancer, cancer cells might interact with fibroblasts to stimulate Notch signaling [69]. However, since squamous cell carcinoma may enhance CAF phenotypes when Notch signaling is absent, this method probably isn’t relevant in all situations [70]. There is a transfer of gastric cancer (GC)-derived exosomal miR-27a to fibroblasts. This transfection leads to a decrease in cysteine and glycine rich protein 2 (CSRP2) expression, an increase in α-SMA expression, and the separation of fibroblasts into CAFs [71]. Pancreatic adenocarcinoma (PAAD) remains one of the most common and lethal tumors. Comparing the tissues of PAAD to the corresponding normal tissues, it was shown that the expression of ACTA2, FAP (fibroblast activation protein), PDGFRα/β (platelet-derived growth factor receptor-α/β), and S100A4 (which is widely used as a marker to detect CAFs) was significantly overexpressed [72]. Numerous inflammatory modulators can activate the CAFs [73]. Interleukin-1 (IL-1) acts via NF-kB and IL-6, and the transcription factor STAT (signal transducer and activator of transcription) [74]. Crosstalk and positive feedback mechanisms such as JAK (Janus kinase)-STAT signaling, the contractile cytoskeleton, and modifications in histone acetylation are the root causes of the further increase in CAF activation [74]. Furthermore, physical alterations occur in the ECM, and CAF is activated [75]. Therefore, tumor cells are the primary source of CAFs, which accelerates the development of tumors.

### The Importance of exosomal miRNAs secreted from CAFs in the spread of cancer and the CAFs

The importance of the microenvironment for the development, maintenance, and spreading of the tumor mass has been mainly shown [76]. The majority of cell types, including cancer cells, release exosomes, which are nanoparticles with a diameter of 30–150 nm that facilitate communication between surrounding cells and tumors [75].

Given that miRNAs are crucial regulators of tumor functionalities, it is becoming more and clearer that alterations driven by cancer govern these miRNA-based networks. A member of the α-arrestin protein family is thioredoxin-interacting protein (TXNIP). It is regarded as a thioredoxin-activity endogenous inhibitor. It has been established that TXNIP had a role in the development of colorectal cancer (CRC). Key components in the formation of CRC are TXNIP and miR-135b-5p from CAFs. CAF exosomes may regulate the production of miR-135b-5p to affect CRC cell proliferation by blocking TXNIP [18].

Unlike other solid tumors, gynecological malignancies have seldom been observed to include exosomes; in particular, endometrial cancer (EC) exosomal miRNAs are thought to play a significant role as a mediator in this two-way communication between cells [77]. However, exosomal miRNAs act as a bridge for information exchange between TAMs, CAFs, and EC cells. They are crucial for the growth of tumor cells, the EMT transition, and ultimately the development of TMEs. Exosomes generated by CAF stimulate the proliferation of EC. Exosomes produced from EC-CAFs may include downregulated miRNAs with tumor suppressor characteristics. Comparatively to NFs, EC- CAFs contained and released substantially less miR-148b in exosomes. DNA methyltransferase 1 (DNMT1) is suppressed when miR-148b translocates to EC cells. In the event that this suppression is not achieved, EMT will rise and EC will get stronger. Accordingly, the downregulation of miR-148b in EC-CAF-derived exosomes contributes to both EC cell invasion and metastasis. Consequently, treating EC with miR-148b overexpression may be therapeutic [78]. Similarly, miR-320a expression in EC cells, EC- CAFs-derived exosomes, and EC-CAFs will decline. MiR-320a suppresses the HIF1α/VEGFA axis, which inhibits the proliferation of EC cells [79]. Poor prognostic indicators such as HIF1α and VEGFA are indicative of EC [63]. Additionally, there is a link between the rise in EC cell radiation sensitivity and the decline in HIF1α/VEGFA levels [80]. Exosomes that overexpress miR-320a may also be employed to treat EC sufferers. Studies on serum exosomal miRNAs and miRNAs produced from CAFs in solid tumors [81, 82] as well as supraglottic laryngeal squamous cell carcinomas (SLSCC) have also been conducted [83, 84]. In patients with SLSCC, exosomal miRNAs generated from CAFs had aberrant expression. Together with their target genes (CCND1, CDKN1B, CDK6, PTEN, and FOS), miR-16-5p, miR-29a-3p, miR-34c-5p, miR-32-5p, and miR-490-5p may form a carcinogenic TME and function as biomarkers for SLSCC therapy [10].

There is evidence that in hepatocellular carcinomas (HCCs), exosomes produced from CAF may control the TME. The wnt/β-catenin signaling pathway is decreased by CAF-derived exosomes containing miR-20a-5p oncogene. It causes the inhibition of actin-binding 1 (LIMA1) in HCC and the inhibition of tumor suppressor LIM domain [85]. GSK3β was effective in β-catenin degradation in prostate cancer (PC). The reason for the growth of PC cells and metastasis through the regulation of GSK3β/β-catenin signaling was actually miR-1290 exosomally secreted CAFs [86]. Exosomes have a crucial function in facilitating the spread of metastatic castration-resistant prostate cancer (CRPC) by transporting proteins such as APOE, LRG1, and ITIH that are directly implicated in tumor growth and the formation of secondary tumors in the bones. The diagnostic relevance of these exosomal proteins in differentiating between CRPC and PC patients is important, and targeting them could be a feasible strategy for CRPC therapy. Moreover, exosomes discharged by PC cells exhibiting diverse androgen response characteristics can impact the formation of new blood vessels in CRPC, with miR-27a-3p being recognized as a crucial mediator in this mechanism. Gaining a comprehensive understanding of how extracellular vesicles contribute to the spread of metastatic CRPC is essential for identifying and developing novel treatment targets for advanced CRPC [87].

The reverse transcriptase telomerase, which lengthens telomeres, is one of the characteristics that set cancer cells apart. This is due to the fact that whereas most somatic cells lack the enzyme, the majority of malignant cells possess this enzyme. Through the upregulation of two CAF markers, α-smooth muscle actin (SMA) and vimentin, exosomal telomerase may aid in the conversion of NFs into CAFs. Telomerase activity modifies the miRNA transcriptomes in these fibroblasts. The proliferative phenotype that these cells adopt after eating exosomal human telomerase reverse transcriptase (hTERT) may be influenced by one of the most well-known miRNAs, miR-342, which is thought to be expressed ectopically as a survival benefit [88]. Moreover, exosomes contain a significant amount of circular RNAs (circRNAs), which have a crucial function in various physiopathological processes including the migration and growth of tumor cells, vascularization in tissues, and the development of tissues and organs. Tumor cells communicate with one another by exchanging exosomal circRNAs, which are signaling molecules, to enhance the growth and progression of tumors. Exosomes have a regulatory impact on tumor formation and change of the tumor microenvironment by carrying circRNAs to tumor cells or other cells in the microenvironment [89]. Exosomal circRNAs play crucial roles in the TME by modulating cancer hallmarks, including angiogenesis, EMT, invasion, and metastasis. They can also regulate anticancer immunity and enhance chemoresistance [89, 90]. Exosomal circRNAs can be used to monitor tumor prognosis and predict postoperative recurrence. For instance, in bladder urethral epithelial carcinoma (UCB), circPRMT5 is overexpressed and positively correlated with low survival in patients. Similarly, a high expression of exosomal circPDE8A is a risk factor for patients with pancreatic ductal adenocarcinoma (PADC), and lower levels of exo-FECR1 are associated with longer disease remissions in small cell lung cancer (SCLC) patients [90]. In bladder cancer, the expression of CircNFIX in serum exosomes is significantly increased in patients with tumor recurrence compared to those with primary tumors [90]. As research on exosomal circRNAs progresses, exosomal circRNAs are anticipated to serve as biomarkers for early detection of bladder cancers and as targeted therapy tools [89]. Exosomal circRNAs have been shown to regulate melanoma progression by modulating the TME. For example, exosomal circRPS5 can inhibit melanoma cell proliferation and invasion [91]. Recent studies have shown that exosomal circRNAs play critical roles in conferring chemotherapy resistance in diverse cancers. Exosomal circRNAs can act as miRNA sponges, interact with RNA-binding proteins, and even encode proteins, thereby modulating the expression of genes involved in drug resistance pathways [92]. In these cancers (lung cancer, gynecological cancers, glioma, breast cancer, prostate cancer, multiple myeloma, and oral squamous cell carcinoma), specific exosomal circRNAs were found to be upregulated and associated with increased proliferation, migration, invasion, and apoptosis resistance in chemotherapy-resistant cancer cells. Mechanistic investigations revealed that these exosomal circRNAs typically regulate chemoresistance by sponging miRNAs and altering the expression of target genes [92].

### The role of CAFs in the resistance to anticancer drugs

Exosomal miRNAs originating from cancer can promote CAF differentiation, and exosomal miRNAs released by CAFs in the TME are essential in the development of therapeutic resistance. Tumor-derived exosomes may have a major impact on the differentiation of CAFs, which increases tumor development, invasive, pro-angiogenic, and drug-resistant phenotypes [34, 93]. Furthermore, Cancer’s response to chemotherapy is controlled by CFAs. Premetastatic lung fibroblasts were similarly stimulated by exosomal miR-1247-3p from HCC cells, which resulted in the overexpression of IL1B, IL-6, and IL-8 as well as resistance to sorafenib therapy [35]. Similarly, exosomal miR-1247-3p from HCC cells triggered CAF in fibroblasts of a lung premetastatic niche. Proinflammatory genes such as IL1B, IL-6, and IL-8 were upregulated as a result, and sorafenib therapy led to the development of therapeutic resistance [35]. Sarfenib is regarded as a targeted therapy for the treatment of advanced HCC, despite its modest effect in terms of overall survival because of drug resistance [94]. In terms of clinical prognosis, sorafenib is unsuccessful due to inflammatory interleukins such as IL-6 [94].

Diverse subtypes of CAFs coexist in pancreatic cancer tissues, where they have the dual ability to accelerate and impede the disease’s progression. Here, cancer cells with p53 mutations that cause GOF (gain of function) give rise to the prevailing population known as CAFs [95]. As a result, it gives p53-null and GOF cancer cells a metastatic environment [95]. GOF mutant p53 cells or their CAFs have the ability to rewire CAFs that were trained by null p53 cancer cells. One important component of this pro-metastatic milieu is perlecan (Heparin sulfate proteoglycan 2, or HSPG2). These dominating CAFs impede the capacity of cancer cells to respond to chemotherapy. Because decreasing perlecan in the stroma in conjunction with chemotherapy boosts mice survival, perlecan is a possible target for anti-stromal treatments in pancreatic cancer. CAFs have the ability to release exosomes carrying miR-20a, which can speed up the development and chemoresistance of non-small-cell lung cancer (NSCLC) [96]. CAF-derived exosomes, especially those that are CD9-positive, inhibit the growth of malignant melanoma cells. The five-year disease-free survival rate was considerably greater in patients who had CD9-positive CAF-made exosomes compared to patients who lacked CD9 exosomes [97]. Tumors with high levels of periostin-positive (POSTN) CAFs significantly decreased the overall survival of the patients [98]. POSTN+CAF showed minimal expression of smooth muscle actin and high rates of protein synthesis and proliferation when they were found in peri-/pre-tumoral regions. They were associated with highly malignant tumors and macrophage infiltrates. The recruitment of dendritic cells and immune-related markers was associated with CAFs that were podoplanin-positive. Specific TME traits were associated with the simultaneous presence of POSTN^+^CAF and podoplanin-positive CAFs, as measured by stromal abundance and immune cell infiltrates. Although the published myofibroblastic CAF (myCAF)/iCAF categorization was unrelated to POSTN^+^CAF, podoplanin-positive CAFs showed a fraction that resembled the inflammatory CAF (iCAF). According to these findings, a POSTN^+^CAF is an early, activated CAF that is associated with aggressive malignancies, whereas a podoplanin-positive CAF is connected to an immune-related phenotype. Together, these two categories establish specific TME and influence patient prognosis; they might be useful for future patient stratification. Targeting or reprogramming “bad” CAF populations (e.g., POSTN) may lead to the development of a novel treatment strategy for pancreatic ductal adenocarcinoma (PDAC) [98]. The platinum-based medication cisplatin creates adducts in the DNA of cancer cells to exert its anticancer effects. These adducts drive cancer cells to undergo apoptosis, or cell death, by inducing the DNA damage response. Cisplatin resistance is a major issue for people with bladder cancer (BC). It has been demonstrated that the delivery of bioactive compounds by exosomes derived from CAFs (CAF-Exo) can enhance chemotherapy resistance in a range of human tumors. Exosomal LINC00355, generated from CAFs, enhances BC cell resistance to cisplatin by modulating the miR-34b-5p/ABCB1 axis [99]. A recent study found that CAF-Exo miR-98-5p downregulates CDKN1A, a crucial regulator of cell cycle arrest, increasing OVCA cell proliferation and fostering a cisplatin-resistant phenotype [100]. Interestingly, several exosomal miRNAs from CAF have been shown to enhance cisplatin resistance. For example, in head and neck cancer cells, CAF-Exo miR-196a reduced the expression of *CDKN1B* (Cyclin Dependent Kinase Inhibitor 1B), a gene critical for the change from the G1 to the S phases of the cell cycle [101]. Furthermore, miR-522, which was produced from CAF exosomes, conferred cisplatin resistance to gastric cancer cells [102]. In the pancreatic TME, CAF-Exo miR-106b was found to directly reduce tumor protein 53-induced nuclear protein 1 (TP53INP1) expression, which in turn increased gemcitabine resistance in pancreatic cancer cells [12]. In patients with PAAD receiving gemcitabine therapy, exosomal miR-146a, which is released by CAF, has also been shown to target *Snail* pathways and accelerate the establishment of the gemcitabine-resistant phenotype [103]. Nano-drug delivery systems (nano-DDSs) can be designed to respond to TME stimuli, enhancing drug retention, accumulation, penetration, and tumor cell uptake [104]. Exosomes have emerged as a promising drug delivery platform for cancer therapies, including melanoma treatment [91, 105]. Exosomes can be engineered to enhance their tumor-targeting capabilities and drug delivery efficiency. Strategies include surface decoration and loading exosomes with therapeutic cargoes [91, 105]. Engineered exosomes can deliver drugs to tumor sites and temporally release therapeutic molecules, improving on the limitations of conventional molecular-targeted drugs [91]. Moreover, nanomaterials used in tumor immunotherapy are into two main categories: organic and inorganic nanomaterials, organic nanomaterials include polymers such as polylactic–co-glycolic acid (PLGA), polyethyleneimine (PEI), polyethylene glycol (PEG), polycaprolactone (PCL), and polyglutamic acid (*γ*-PGA), as well as cell membrane-derived structures like tumor cell membranes, macrophage membranes, platelet membranes, and erythrocyte membranes. These organic nanomaterials exhibit advantageous properties like biocompatibility but may face challenges such as instability and rapid metabolism. In contrast, inorganic nanomaterials like graphene, black phosphorus, and silicon demonstrate enhanced stability and drug delivery capabilities but potentially pose greater toxicity concerns [106]. Lili Cheng et al. designed and created a therapeutic nanovesicle known as hGLV. Cheng et al. developed a hybrid therapeutic nanovesicle, named hGLV, by combining genetically modified exosomes with drug-containing thermosensitive liposomes. The overexpression of CD47 in hGLV was seen to modify the process of tumor cell phagocytosis by macrophages through the inhibition of CD47 signaling [107] Nanoparticle-enhanced radiotherapy is significant in triggering robust cancer immunotherapy because it allows for the combination of antitumor drugs to kill tumors while also enhancing the body’s immunity. This multi-treatment effect is achieved through the use of multifunctional nanomaterials, which can overcome the limitations of traditional immunotherapy strategies such as low bioavailability, low response rates, and severe side effects. By utilizing nanotechnology, researchers have been able to break through the bottleneck problem of antitumor immunotherapy, offering new possibilities for more effective cancer treatment [108].

## Discussion

The CAFs within the TME interact with cancer cells in ways that are essential to the growth and dissemination of disease. It is now recognized that within the TME, CAFs, and myofibroblasts are extremely diverse cells with unique gene expression patterns and a variety of biological roles that often contradict one another [72] (Fig. 1). Multiple CAF subpopulations may be seen inside a single tumor. CAFs partly mediate their actions by altering the ECM and secreting soluble components and EVs. EVs, of which exosomes are a subclass, are microscopic sacs that contain a range of biomolecules, such as proteins, nucleic acids, and lipids. Exosomes transport a payload of lipids, proteins, and nucleic acids that resemble the biological makeup of the cells from which they were derived. The tumor’s malignant nature is regulated by its surroundings. The TME is composed of biological components such as tumor cells, CAFs, endothelium, and immunological cells, as well as non-cellular components such as exosomes and cytokines, which all play important roles [109]. CAFs are stromal cells within the TME that have been found to promote tumor growth, invasion, and metastasis. They play a significant role in creating a supportive environment for cancer cells by secreting various factors that aid in tumor progression. In particular, CAF-derived exosomes have been implicated in mediating communication between CAFs and cancer cells, thereby promoting tumor progression and therapy resistance. These exosomes may contain specific bioactive molecules that contribute to the crosstalk within the TME, ultimately impacting tumor growth and response to therapy [92]. CAF secretes proinflammatory cytokines such as IL-1 and IL-8, both of which have pro-tumor effects. According to studies, tumor-derived exosome miRNA-1247-3p can activate tumor-associated fibroblasts, causing them to produce cytokines such as IL-6 and IL-8, which promotes lung metastasis of liver cancer [35]. Numerous investigations have revealed that exosome-associated miRNA-9 and miRNA-200s enhance metastasis and the transformation of NFs into CAFs [36]. Multiple vascular supports are required for cancer progression and can provide nourishment and growth factors to tumors. Exosomes released in low oxygen conditions augment the stem cell characteristics in Ewing’s sarcoma by delivering a concentrated amount of miR-210 that suppresses the apoptotic pathway, resulting in cell viability and promoting the creation of cellular spheres. In the microenvironment of bone sarcomas, EVs have a vital function in facilitating communication between different cells. Additionally, they can be used as biomarkers to aid in the diagnosis and prognosis of these conditions. An analysis is conducted on the role of EVs in facilitating communication between various cells inside the microenvironment of bone sarcomas. This research yields novel insights that can aid in the identification of therapeutic targets and diagnostic analysis [110].

Previous studies have demonstrated that exosomal miRNAs released by tumor cells have a significant influence on vasculature remodeling via IL-8-activated VCAM-1. Exosome-associated miRNA-526b and miRNA-655 also increase lymphangiogenesis and angiogenesis [111], whereas miRNA-340-5p and miRNA-561 promote an immunosuppressive TME [112, 113]. Exosomes produced from CAFs have been demonstrated to greatly promote OVCA tumor growth. CAFs directly transported exosomes into OVCA cells to boost intracellular circIFNGR2 levels. Exosomal circIFNGR2 activation inhibited cell proliferation, metastasis, and EMT. Mechanistically, elevated circIFNGR2 activated the miR-378/ST5 axis, preventing tumor cells from evolving into malignant cells [109]. The methods by which CAFs influence PC tumorigenesis remain unknown. When CAFs-Exo was compared to NFs-Exo, the level of miR-1290 was considerably greater. CAFs dramatically improved PC cell motility, invasion, stemness, and metastasis by transmitting exosomal miR-1290 [114]. The lack of precise CAF markers substantially limits depletion techniques. Nonetheless, CAF depletion was the first technique to be shown as a supplement to immunotherapies. Reducing FAzP (fibroblast activation protein)^+^ CAFs enhanced the anticancer vaccine’s efficacy, according to seminal research by Kraman et al. [115]. Recent research has identified CAF-mediated CXCL12 expression as a defining characteristic of the “CAF-S1” immunosuppressive subtype of breast cancer myofibroblastic CAF (myCAF) [116]. Pre-clinical studies have also shown that the clinically approved inhibitor AMD3100 is effective in blocking the interaction between CXCL12 and its cognate receptor CXCR4 [117, 118]. The complex and intricate signaling network that involves TGF-β, PI3Ks/AKT/mTOR (Mammalian target of rapamycin), MAPK (Mitogen-activated protein kinase), Wnt, Janus kinase/signal transducers and activators of transcription, EGFR (Epidermal growth factor receptor), Hippo, and nuclear factor kappa-light-chain-enhancer of activated B cells, among other signaling pathways, makes CAFs vulnerable to crosstalk with cancer cells. Exosomes carrying miRNA are released by CAFs, healthy fibroblasts, and cancer cells that form a network of cell-to-cell communication in the TME. These CAF signals reveal distinct characteristics as the disease advances and may be the target of anticancer therapy.

## Conclusion

CAFs are essential elements of the TME and play an important role in the interaction between cancer cells and their environment. An essential function of cancer cells is to mediate the development and activation of CAFs. Furthermore, the development of cancer is dependent on both CAF activation and malignant cancer cells. Oncological behaviors are caused by crosstalk between cancer cells and CAFs. Within the TME, NFs, cancer cells, and CAFs all release miRNA-containing exosomes that facilitate cell-to-cell communication. Consequently, CAFs require a focused treatment that improves anticancer therapy in both in vivo and in vitro investigations. MiRNAs are involved in cancer cells as well as the tumor microenvironment. According to the body of data, the relationship between tumor cells and the TME may be impacted by the dysregulation of miRNAs and ex-miRNAs. Thus, it is anticipated that removing CAFs will make it easier to cure cancer and prevent cancer cells from metastasizing and developing resistance. However, a major barrier to more readily treating cancer with therapeutic techniques is the absence of precise markers for CAFs.
